# PSYCHOSOCIAL RESILIENCE, FAMILY SUPPORT, AND COGNITIVE STATUS: FROM EVIDENCE TO ACTION

**DOI:** 10.1093/geroni/igac059.067

**Published:** 2022-12-20

**Authors:** Hanzhang Xu, Bei Wu, Man Guo

## Abstract

Increasing evidence suggests the important role of social connections and family support in maintaining optimal cognitive status among older adults. This symposium includes four studies from China and the U.S with a focus on generating actionable evidence to inform the development of strategies that target psychosocial resilience and family support to promote cognitive health. Using data from the 2006, 2010, and 2014 waves of the Health and Retirement Study, the first study assessed the impact of social isolation on cognitive function, and how sleep disturbance mediated the association on cognitive decline. The study findings suggest addressing sleep disturbance might be a viable way to mitigate the negative effect of social isolation on cognitive function. Companion piece includes another HRS-based study that assessed the impact of loneliness on psychological resilience and cognitive health in later life. Findings from this study show loneliness is indirectly associated with baseline cognitive status and accelerated cognitive decline through deteriorating phycological resources. The third study used a prospective longitudinal design and applied group-based trajectory modeling to identify distinct family functions among 170 Chinese stroke survivors. Four family function trajectories were identified; healthy and stable family function was associated with better cognition and quality of life. Lastly, the fourth study aimed to use an experienced-based co-design approach to develop a cognitive training intervention to promote cognitive health in older Chinese immigrants in the U.S. This approach allows researchers to engage end-users early and to optimize the development of a culturally and linguistically relevant cognitive training intervention.

